# High intake of dietary phytochemical index may be related to reducing risk of diabetic nephropathy: a case–control study

**DOI:** 10.1186/s40795-023-00676-2

**Published:** 2023-01-16

**Authors:** Niki Bahrampour, Atieh Mirzababaei, Dorsa Hosseininasab, Faezeh Abaj, Cain C. T. Clark, Khadijeh Mirzaei

**Affiliations:** 1 Department of Nutrition, Science and Research Branch, Islamic Azad University (SRBIAU), Tehran, Iran; 2 Department of Community Nutrition, School of Nutritional Sciences and Dietetics, Tehran University of Medical Sciences (TUMS), P.O. Box 14155-6117, Tehran, Iran; 3 Centre for Intelligent Healthcare, Coventry University, Coventry, CV1 5FB UK

**Keywords:** Phytochemical index, Diet, Diabetic nephropathy, Diabetes

## Abstract

**Objective:**

Diabetic nephropathy (DN) is involved in 40% of patients with type 2 diabetes, Phytochemical index (PI) foods are known as antioxidant and anti-inflammatory agents. Higher intake of phytochemicals can improve glucose tolerance, hypertension and complications of DN. This study sought to discern the relationship between dietary PI and DN.

**Methods:**

This was a case–control study which was conducted between 210 diabetic women. General characteristics, blood pressure, biochemical serum levels, and anthropometric measurements were evaluated. Physical activity and dietary intakes were assessed via short form of physical activity questionnaire and 147 items-validated food frequency questionnaires, respectively. Then, PI was calculated through method of McCarty and divided to 2 groups of lower and higher of median. Independent samples T tests were used to identify differences in quantitative variables. To investigate the relationship between dietary PI and risk of DN, logistic regression was used. The odds ratio (OR) of DN, and its 95% confidence interval (CI), in each groups of PI were shown.

**Results:**

The percentage of daily intake of energy from fruits and vegetables were higher than the other sources of phytochemical rich foods. Higher consumption of vitamin A was seen in higher group of PI among the control group, after adjusting for energy intake. In the higher adherence of median of dietary PI group, intake of fruits, vegetables, legumes, grains, and olives of controls were higher than cases. In addition, soy consumption was statistically different between lower and higher adherence of median of dietary PI among cases. There was an inverse relationship between dietary PI and risk of DN (OR = 0.44; 95% CI: 0.25–0.77; *P* = 0.04). After adjusting for potential confounders, the association remained significant, albeit with lower odds of having DN (OR = 0.15; 95% CI: 0.06–0.36; *P* < 0.001).

**Conclusion:**

Finally, the present study found evidence indicating an inverse relationship between consumption of foods rich in phytochemicals and risk of DN in this sample.

**Supplementary Information:**

The online version contains supplementary material available at 10.1186/s40795-023-00676-2.

## Highlights

• Higher intakes of phytochemical rich foods may be associated with lower risk of diabetic nephropathy.

• Consumption of vitamin A, fruits, vegetables, legumes, grains, and olives may prevent nephropathy among patients with diabetes.

**Supplementary Information:**

The online version contains supplementary material available at 10.1186/s40795-023-00676-2.

## Introduction

Type 2 diabetes mellitus (DM) is a metabolic disorder, characterized by non-insulin dependent hyperglycemia. Polyuria, polydipsia, blurred vision and feeling tired are the common signs of the disorder; whilst uncontrolled hyperglycemia and hyperinsulinemia may alter organs function, including kidney, eyes, and nerves [1]. Diabetic kidney disease is involved in around 40% of patients with type 2 diabetes. The global diabetes prevalence in 2019 is estimated to be 9.3% (463 million people) and 10.9% (700 million) by 2045 [2]. Indeed, diabetes is the ninth direct cause of death in women in the world, and women with type 2 diabetes are more susceptible to not being treated than male counterparts [3]. Impaired endothelial integrity, microalbuminuria, and impairment of nitric oxide transport, and loss of glomerular filtration capabilities are seen in diabetic nephropathy (DN) [4]. Older age, sex (men), race/ethnicity, family history of DN, genetic, hypertension, kidney injuries, toxins and smoking can be risk factors for DN [5], whilst one of the most significant factors that may be related with DN is dietary intakes [6]. An observational study showed that adherence to the Mediterranean diet which included a high consumption of monounsaturated fat by using olive oil was associated with lower risk of kidney function decline. Another dietary strategy which can postpone developing blood pressure and DM is Approaches to Stop Hypertension (DASH). Higher intake of vegetables, whole grains, and fruit and plant proteins (e.g., nuts, seeds, and beans) and its components (e.g., potassium or isoflavones) may have this effect [6].

Phytochemicals are bioactive substances derived from plants, which are abundant in fruits, vegetables, legumes, whole grains, and nuts [7]. Studies have shown many positive benefits for human health, especially metabolic disorders; for instance, an inverse association between phytochemical intakes and prediabetes was reported in Kim et al. [8]. Polyphenols, phytoestrogens, and organosulfur and plant sterols, which we term dietary phytochemicals, can help control blood glucose, hypertension, and insulin level [7]. Phytochemicals are known as antioxidant and anti-inflammatory agents, and previous studies have suggested that consumption of different phytochemicals can help control fasting blood glucose in healthy and diabetic patients [9]. Polyphenols regulate insulin production, fight against oxidative stress in pancreatic β-cells, induce the cell membrane localization of GLUT4 via the activation of AMP-activated protein kinase. Quercetin, a flavonoid rich in onion, have play a role in reducing blood pressure, visceral fat, promoting the apoptosis of adipocytes, while genistein, a flavonoid high in soy products, is associated with decreases in body fat mass and increases in high density lipoprotein (HDL) and glucose tolerance [10]. Phytochemical index (PI), incepted by McCarty, is defined as a percentage of calories intake derived from food rich in phytochemicals [11]. Flavonoids, which are a part of PI, can enhance endothelial cell function; in addition, olives, legumes, fruits, and vegetables (subgroups of PI) can improve hypertension, which is directly associated with nephron health [9]. According to past studies, a higher PI is associated with lower accumulation of fat especially in waist circumference, blood pressure, and lipid profiles level. Overall, higher intake of phytochemicals from foods can improve complications of DN [8].

Based on the high prevalence of DN in women in Iran, it is necessary to discern dietary factors that may be associated with this disease [12]. Given that no study has investigated the relationship between PI and DN, the aim of this study was to investigate the association between dietary PI and DN among Iranian women.

## Materials and methods

### Population

This case–control study was conducted in Kowsar Diabetes Clinic in Semnan, Iran in 2016. For both case and control groups, participants were included if they were women, diagnosed type 2 diabetes, aged between 30–65 years, fasting blood glucose (FBG) ≥ 126 mg/dl, or 2-h post-load blood glucose (2hrBG) ≥ 200 mg/dl; glycosylated hemoglobin (HbA1c) ≥ 6.5% for 3–10 years [13]. Women with any chronic diseases, such as hepatic disease, coronary angiography, stroke, cancer, autoimmune disorders, etc. were excluded. The pregnant and lactating women were excluded. The medical history of patient was asked too. Half of the participants who were in the case group had diabetic nephropathy (DN). DN is diagnosed by persistent albuminuria on two or more occasions, separated at least by three months on early morning urine samples [14]. Urinary albumin level was calculated in a random spot urine sample by enzyme-linked immunosorbent assay [sensitivity 0.001 mg/L; coefficient of variation (CV) 4.5e7.6%]. Urinary mg of albumin per gram of creatinine (ACR) ≥ 30 mg/g was discerned in a random spot urine sample [15]. According to the patient’s medical history, the patients had glomerular filtration rate < 60. 105 women were chosen as cases with DN. 105 controls were selected by a 1:1 matching to 105 cases by age at 1-year intervals and diabetes duration at 6-month intervals without DN. General characteristics of subjects were collected via a demographic questionnaire, whilst systolic and diastolic blood pressure was evaluated via sphygmomanometer.

### Biochemical measurements

Blood levels of FBG, 2HBG, HbA1C were measured, whilst other biochemical markers, including triglycerides (TG), low and high-density lipoprotein (LDL, HDL), total cholesterol (TC), serum creatinine (Cr), and blood urea nitrogen (BUN), were collected from their medical records from the last three months.

### Anthropometric and physical activity measurements

Weight (kg) and height (cm) were measured, with participants in light clothing and unshod, using Seca 216 to the nearest 0.1 cm and a digital scale (SECA, Hamburg, Germany) to the nearest 0.1 kg, respectively, by an expert dietitian. Waist circumference (WC) (cm) was measured, and body mass index (BMI) (kg/m^2^) was calculated according to standard formulae. Physical activity (PA) was assessed by completing the validated short form of the international physical activity questionnaire (IPAQ) [16, 17].

### Dietary intakes and phytochemical index

To discern the dietary intakes of participants, the 147 items-validated food frequency questionnaire (FFQ) was used. Total foods intakes were converted to gram per day [18]. NUTRITIONIST 4 (First Data Bank, San Bruno, CA) software was used to evaluate the nutrients content. The dietary phytochemical index (PI) was calculated through method of McCarty; [PI = (daily energy derived from phytochemical-rich foods kcal/total daily energy intake kcal) × 100] [11]. Phytochemical-rich foods included fruits and vegetables (except potatoes), their juices like tomato sauces, legumes, whole grains, nuts, soy products, olives, and olive oil [19]. Total energy intake of < 500 or > 3500 kcal was accepted.

### Statistical analysis

The normality of data was evaluated using visual graph inspection and the Kolmogorov–Smirnov test. Qualitative and quantitative variables were shown as frequency (%) and mean ± SD respectively. Dietary PI was divided to two groups according to the median (lower and higher adherence). General characteristics of participants were compared among case and control groups and PI groups using an independent sample T test and Chi-square test. Dietary intakes of the participants across PI groups were assessed using general linear models and independent sample T test. To investigate the relationship between dietary PI and DN, logistic regression was used. The odds ratio (OR) of DN, and its 95% confidence interval (CI), was shown for each groups of PI. Two models were created: Model 1 was adjusted for age, BMI, energy intake, and physical activity (PA). Model 2 was adjusted for confounders in model 1, plus diabetes duration, cardiovascular diseases history, and drug usage (angiotensin receptor blockers; angiotensin converting enzyme inhibitors, beta-blockers, metformin, sulphonyl urea, and insulin). All statistical analysis were conducted using SPSS (Version 16.0; SPSS Inc., Chicago, IL), and *p* values < 0.05 were considered significant.

## Results

### General characteristics of participants

General characteristics of the subjects across case and controls, and groups of PI, were shown in Tables 1 and 2. The mean ± SD of age in group of case were 55.33 ± 7.04 (years), whilst the mean ± SD of FBS and serum creatinine, albumin, and dietary PI in (case and control) groups were (167.10 ± 50.62–154.19 ± 45.03) (mg/dl), (0.92 ± 0.16–0.87 ± 0.17) (mg/dl), (14.40 ± 11.94–8.37 ± 6.76) (mg/dl) and (88.01 ± 29.96–103.28 ± 43.83), respectively. Angiotensin receptor blockers (ARBs) and angiotensin converting enzyme inhibitors (ACEIs) prescription was significantly different between cases and controls (*P* < 0.05). In addition, in the case group, those in the higher adherence of median of dietary PI had a lower level of FBS (*P* = 0.04). In the control group those in higher adherence of median of dietary PI had a lower level of albumin (*P* = 0.001).

**Table 1:** General characteristics of subjects across case and control groups

Variables	Control (*n* = 105)	Case (*n* = 105)	*P* value*
**Age (y)**	55.41 ± 7.14	55.33 ± 7.04	0.94
**Albumin (g/dl)**	8.37 ± 6.76	14.40 ± 11.94	** < 0.001**
**ACR**	18.66 ± 5.92	232.18 ± 114.07	** < 0.001**
**Diabetes duration (y)**	7.56 ± 2.17	7.60 ± 2.21	0.88
**SBP (mmHg)**	129.04 ± 98.88	126.59 ± 17.27	0.80
**DBP (mmHg)**	80.10 ± 11.76	82.80 ± 13.09	0.12
**Body weight (kg)**	71.589 ± 11.50	73.400 ± 13.83	0.30
**Height (cm)**	161.17 ± 5.91	160.68 ± 6.29	0.56
**BMI (kg/m**^**2**^**)**	27.510 ± 4.39	28.686 ± 4.74	0.06
**Hb (mg/dl)**	12.630 ± 1.22	12.610 ± 1.37	0.91
**FBS (mg/dl)**	154.19 ± 45.03	167.10 ± 50.62	**0.03**
**BS (mg/dl)**	207.10 ± 54.35	217.75 ± 53.23	0.15
**HbA1c (%)**	8.031 ± 1.29	8.660 ± 1.41	** < 0.001**
**Cholesterol (mg/dl)**	175.38 ± 32.42	185.15 ± 38.12	**0.05**
**TG (mg/dl)**	162.25 ± 57.91	167.26 ± 65.68	0.56
**LDL (mg/dl)**	94.60 ± 29.47	106.86 ± 31.77	** < 0.001**
**HDL (mg/dl)**	46.37 ± 9.25	45.05 ± 9.26	0.30
**Cr (mg/dl)**	0.87 ± 0.17	0.92 ± 0.16	**0.03**
**BUN (mg/dl)**	15.17 ± 3.86	15.79 ± 4.55	0.29
**Energy intake (kcal)**	1452.26 ± 320.95	1407.64 ± 254.51	0.27
**PI**	103.28 ± 43.83	88.01 ± 29.96	** < 0.001**
**PA (met-h/w)**			
Low (> 600)	37 (17.6)	31 (14.8)	0.12
Moderate (600–3000)	28 (13.4)	42 (20)	
High (< 3000)	40 (19)	32 (15.2)	
**Medical history**			
** CVD history**	23 (11)	24 (11.4)	0.86
**Medication usage**			
** ARBs**	45 (21.4)	60 (28.6)	**0.03**
** ACEIs**	21 (10)	44 (21)	**0.001**
** Beta blockers**	18 (8.6)	20 (9.5)	0.56
** Metformin**	104 (49.5)	104 (49.5)	0.75
** sulfonylurea**	62 (29.5)	71 (33.8)	0.19
** Insulin**	35 (16.7)	26 (12.4)	0.17

Independent sample T test and chi square were used

Continuous variables were shown as mean ± SD and qualitative variables were shown n (%)

*Abbreviation: ACR* Albumin creatinine ratio, *SBP* Systolic blood pressure, *DBP* Diastolic blood pressure, *BMI* Body mass index, *Hb* Hemoglobin, *FBS* Fasting blood sugar, *BS* Blood sugar, *TG* Triglycerides, *HDL* High density lipoprotein, *LDL* Low density lipoprotein, *CR* Creatinine, *BUN* Blood urea nitrogen, *PI* Phytochemical index, *PA* Physical activity, *met* metabolic equivalent, *CVD* Cardiovascular disease, *ARBs* Angiotensin receptor blockers, *ACEIs* Angiotensin converting enzyme inhibitors

^*^ Adjusted for energy intake

*P* < 0.05 was considered significant

**Table 2:** General characteristics of subjects between lower and higher adherence of dietary phytochemical index

Variables	PI					
	**Control**			**Case**		
	**Low(*n* = 52)**	**High(*n* = 53)**	*P* value*	**Low(*n* = 52)**	**High(*n* = 53)**	*P* value^*^
**Age (y)**	54.92 ± 6.59	55.89 ± 7.67	0.18	54.75 ± 7.64	55.91 ± 6.41	0.08
**Albumin (g/dl)**	11.19 ± 8.00	5.61 ± 3.61	** < 0.001**	16.27 ± 13.40	12.57 ± 10.11	0.98
**Diabetes duration (y)**	7.85 ± 2.13	7.27 ± 2.19	0.62	7.60 ± 2.19	7.61 ± 2.24	0.65
**SBP (mmHg)**	121.08 ± 16.11	136.85 ± 138.47	0.18	127.71 ± 16.59	125.49 ± 17.99	0.31
**DBP (mmHg)**	80.69 ± 12.23	79.51 ± 11.36	0.42	82.85 ± 14.34	82.75 ± 11.87	0.36
**Body weight (kg)**	70.21 ± 11.85	72.95 ± 11.09	0.58	75.17 ± 12.78	71.66 ± 14.70	0.76
**Height (cm)**	161.71 ± 5.91	160.64 ± 5.92	0.94	161.06 ± 6.67	160.30 ± 5.93	0.44
**BMI (kg/m**^**2**^**)**	26.78 ± 4.42	28.22 ± 4.28	0.80	29.03 ± 5.03	28.35 ± 4.47	0.49
**Hb (mg/dl)**	12.51 ± 1.23	12.75 ± 1.22	0.88	12.43 ± 1.38	12.78 ± 1.34	0.67
**FBS (mg/dl)**	158.35 ± 50.40	150.11 ± 39.11	0.26	178.42 ± 57.17	156.00 ± 40.81	**0.04**
**BS (mg/dl)**	208.27 ± 53.30	205.94 ± 55.85	0.60	228.85 ± 61.07	206.87 ± 42.01	**0.02**
**HbA1c (%)**	8.23 ± 1.31	7.84 ± 1.25	0.69	8.97 ± 1.51	8.35 ± 1.24	0.40
**Cholesterol (mg/dl)**	177.31 ± 29.18	173.49 ± 35.48	0.54	188.50 ± 35.37	181.87 ± 40.71	0.17
**TG (mg/dl)**	158.69 ± 60.58	165.74 ± 55.51	0.34	176.77 ± 65.21	157.92 ± 65.42	0.30
**LDL (mg/dl)**	98.63 ± 28.61	90.64 ± 30.03	0.88	109.77 ± 32.70	104.00 ± 30.87	0.77
**HDL (mg/dl)**	47.58 ± 10.05	45.19 ± 8.32	0.07	44.92 ± 8.80	45.17 ± 9.77	0.45
**Cr (mg/dl)**	0.85 ± 0.15	0.90 ± 0.19	0.23	0.95 ± 0.17	0.89 ± 0.15	0.65
**BUN (mg/dl)**	15.50 ± 3.95	14.85 ± 3.78	0.42	15.78 ± 4.27	15.81 ± 4.84	0.45
**PA (met-h/w)**						
Low (> 600)	20 (19)	17 (16.2)	0.78	13 (12.4)	18 (17.1)	0.41
Moderate (600–3000)	13 (12.4)	15 (14.3)		24 (22.9)	18 (17.1)	
High (< 3000)	19 (18.1)	21 (20)		15 (14.3)	17 (16.2)	
**Medical history**						
** CVD history**	14 (13.3)	9 (8.6)	0.21	14 (13.3)	10 (9.5)	0.32
**Medication usage**						
** ARBs**	24 (22.9)	21 (20)	0.49	31 (29.5)	29 (27.6)	0.61
** ACEIs**	11 (10.5)	10 (9.5)	0.77	24 (22.9)	20 (19)	0.38
** Beta blockers**	12 (11.4)	6 (5.7)	0.11	14	6 (5.7)	0.08
** Metformin**	51 (48.6)	53 (50.5)	0.49	51 (48.6)	53 (50.5)	0.49
** sulfonylurea**	30 (28.6)	32 (30.5)	0.78	35 (33.3)	36 (34.3)	0.94
** Insulin**	19 (18.1)	16 (15.2)	0.49	15 (14.3)	11 (10.5)	0.33

Independent sample T test and chi square were used

Continuous variables were shown as mean ± SD and qualitative variables were shown n (%)

*Abbreviations: SBP* Systolic blood pressure, *DBP* Diastolic blood pressure, *BMI* Body mass index, *Hb* Hemoglobin, *FBS* Fasting blood sugar, *BS* Blood sugar, *TG* Triglycerides, *HDL* High density lipoprotein, *LDL* Low density lipoprotein, *CR* Creatinine, *BUN* Blood urea nitrogen, *PI* Phytochemical index, *Pa* Physical activity, *met* metabolic equivalent, *CVD* Cardiovascular disease, *ARBs* Angiotensin receptor blockers, *ACEIs* Angiotensin converting enzyme inhibitors

^*^ Adjusted for energy intake

*P* < 0.05 was considered significant. Low and High are presented as lower and higher adherence of median

### Dietary intakes of participants

The relative distribution of different phytochemical rich components of PI score and total nutrients intakes across PI groups were shown in Table 3. The percentage of daily intake of energy from fruits and vegetables were higher than other sources of phytochemical rich foods, and the percent share of olives was the least among phytochemical rich foods. Dietary cholesterol and sodium were lower in higher adherence of median of PI score among the control group (*P* < 0.005). Conversely, higher consumption of vitamin A was seen in the higher group among the control group after adjusting energy intake. Intakes of fruits, vegetables, legumes, grains, and olives were higher among controls in low and high groups of PI. Sodium, fat, and carbohydrate consumption were significantly lower in higher adherence of median of PI in both cases and controls (*p* < 0.05), after adjusting for energy intake. Finally, soy consumption was statistically significantly different between PI groups in cases (*P* = 0.01).

**Table 3:** Dietary intakes of participants between groups of phytochemical index

Variables Amounts per day	PI score							
	**Control**				**Case**			
**Nutrients**	**Low**	**High**	***P* value**	*P* value*	**Low**	**High**	***P* value**	*P* value^*^
**Energy intake (kcal)**	1684.61 ± 282.04	1224.30 ± 145.08	** < 0.001**	-	1534.18 ± 281.72	1283.50 ± 141.42	** < 0.001**	-
**Protein (g)**	53.78 ± 9.79	42.70 ± 6.83	** < 0.001**	0.17	49.12 ± 9.57	42.52 ± 4.60	** < 0.001**	0.34
**Carbohydrate (g)**	302.52 ± 65.79	211.54 ± 23.99	** < 0.001**	0.22	269.03 ± 42.45	223.40 ± 27.26	** < 0.001**	**0.003**
**Fat (g)**	36.07 ± 6.51	29.13 ± 5.28	** < 0.001**	0.17	36.18 ± 11.48	30.62 ± 4.20	**0.001**	**0.01**
**Fiber (g)**	43.56 ± 11.99	34.87 ± 4.58	** < 0.001**	0.28	39.30 ± 7.52	36.06 ± 6.01	**0.01**	0.85
**Cholesterol (g)**	11.22 ± 10.42	5.40 ± 9.67	**0.004**	**0.04**	5.98 ± 5.05	4.42 ± 4.20	0.08	0.49
**SFA (g)**	7.00 ± 1.40	5.51 ± 1.25	** < 0.001**	0.48	6.81 ± 2.10	5.55 ± 1.10	** < 0.001**	0.21
**MUFA (g)**	12.15 ± 2.81	9.72 ± 2.01	** < 0.001**	0.66	12.42 ± 4.32	9.97 ± 1.67	** < 0.001**	0.23
**Na (mg)**	3670.81 ± 829.25	3125.01 ± 987.77	**0.003**	**0.002**	4135.84 ± 1155.69	3182.96 ± 617.43	** < 0.001**	**0.001**
**Potassium (mg)**	1899.36 ± 531.52	1550.45 ± 199.48	** < 0.001**	0.11	1810.21 ± 389.59	1576.70 ± 238.07	** < 0.001**	0.43
**Vitamin C (mg)**	13.85 ± 7.03	8.31 ± 6.07	** < 0.001**	0.28	11.10 ± 4.27	8.43 ± 3.14	** < 0.001**	**0.003**
**Calcium (mg)**	455.36 ± 65.33	361.72 ± 59.07	** < 0.001**	0.29	430.11 ± 69.46	369.71 ± 51.14	** < 0.001**	0.49
**Fe (mg)**	16.63 ± 2.49	13.41 ± 1.65	** < 0.001**	0.78	15.81 ± 2.32	13.80 ± 1.31	** < 0.001**	0.36
**Vitamin E (mg)**	5.08 ± 2.26	3.38 ± 0.64	** < 0.001**	0.18	4.19 ± 1.11	3.66 ± 0.66	**0.003**	0.82
**Thiamin (mg)**	1.94 ± 0.39	1.49 ± 0.21	** < 0.001**	0.29	1.79 ± 0.30	1.54 ± 0.16	** < 0.001**	0.08
**Riboflavin (mg)**	1.10 ± 0.21	0.85 ± 0.12	** < 0.001**	0.19	1.04 ± 0.20	0.89 ± 0.09	** < 0.001**	0.42
**Niacin (mg)**	18.20 ± 3.07	14.00 ± 2.05	** < 0.001**	0.40	17.35 ± 2.90	14.77 ± 1.58	** < 0.001**	**0.02**
**B6 (mg)**	0.80 ± 0.16	0.71 ± 0.11	**0.002**	0.99	0.84 ± 0.15	0.73 ± 0.09	** < 0.001**	**0.04**
**Folate (μg)**	464.15 ± 136.70	341.85 ± 34.96	** < 0.001**	0.17	390.29 ± 72.40	341.37 ± 44.33	** < 0.001**	0.53
**B12 (μg)**	0.22 ± 0.17	0.12 ± 0.19	**0.004**	0.06	0.14 ± 0.09	0.10 ± 0.08	**0.02**	0.89
**Vitamin K (μg)**	14.70 ± 6.34	11.71 ± 1.89	**0.001**	0.49	14.42 ± 6.72	11.58 ± 2.14	**0.004**	0.41
**Vitamin A (RAE)**	22.65 ± 16.15	22.89 ± 12.61	0.93	**0.05**	25.16 ± 12.23	19.33 ± 11.56	**0.01**	0.15
**Phosphorus (mg)**	999.78 ± 163.47	824.09 ± 139.27	** < 0.001**	0.38	965.77 ± 161.53	840.60 ± 117.28	** < 0.001**	0.22
**Magnesium (mg)**	374.58 ± 71.44	331.65 ± 58.95	**0.001**	0.43	383.47 ± 98.41	333.89 ± 50.03	**0.001**	0.79
**Zinc (mg)**	8.67 ± 1.53	7.68 ± 1.39	**0.001**	0.11	8.73 ± 3.77	7.69 ± 1.13	**0.05**	0.11
**Copper (mg)**	1.65 ± 0.28	1.44 ± 0.18	** < 0.001**	0.77	1.62 ± 0.33	1.47 ± 0.12	**0.003**	0.19
**Manganese (mg)**	8.37 ± 1.74	7.55 ± 1.79	**0.01**	0.91	8.95 ± 1.71	7.80 ± 1.41	** < 0.001**	0.01
**Chromium (mg)**	0.22 ± 0.07	0.21 ± 0.08	0.44	0.67	0.24 ± 0.07	0.21 ± 0.07	**0.02**	0.20
**Selenium (μg)**	134.21 ± 33.35	108.80 ± 22.73	** < 0.001**	0.81	133.48 ± 24.16	111.93 ± 18.73	** < 0.001**	**0.01**
**PI components**								
** Caffeine (mg)**	150.21 ± 79.47	135.20 ± 68.09	0.30	0.35	177.46 ± 104.76	147.61 ± 93.34	0.13	0.12
** Coffee (mg)**	13.62 ± 48.50	9.51 ± 42.69	0.64	0.91	16.56 ± 52.88	0.92 ± 1.60	**0.03**	0.65
** Soy (g)**	24.41 ± 32.66	21.08 ± 12.56	0.49	0.99	6.83 ± 14.88	14.44 ± 14.27	**0.009**	**0.01**
** Spices (g)**	2.91 ± 1.89	4.15 ± 3.60	**0.03**	0.75	2.87 ± 2.01	3.04 ± 2.10	0.67	0.22
** Tea (mg)**	750.24 ± 422.56	684.67 ± 391.47	0.41	0.86	879.90 ± 554.44	730.20 ± 465.82	0.13	**0.02**
** Olive (g)**	0.79 ± 1.43	1.45 ± 1.51	**0.02**	**0.02**	0.37 ± 0.91	1.12 ± 1.32	**0.001**	**0.003**
** Nuts (g)**	24.38 ± 7.58	28.73 ± 13.28	**0.04**	**0.02**	27.87 ± 36.95	26.78 ± 9.99	0.83	** < 0.04**
** Grains (g)**	64.79 ± 86.07	72.25 ± 110.87	0.70	**0.001**	35.95 ± 64.74	67.99 ± 96.03	**0.04**	**0.002**
** Legumes (g)**	94.92 ± 74.51	103.30 ± 52.05	0.50	**0.03**	48.49 ± 24.41	67.61 ± 36.30	**0.002**	**0.001**
** Vegetables (g)**	317.90 ± 109.97	454.98 ± 157.41	** < 0.001**	** < 0.001**	214.82 ± 104.18	364.57 ± 270.65	** < 0.001**	** < 0.001**
** Fruits (g)**	391.60 ± 127.72	622.51 ± 292.64	** < 0.001**	** < 0.001**	303.84 ± 97.04	497.08 ± 254.00	** < 0.001**	** < 0.001**

Independent sample T-test was used

*Abbreviation: PI* Phytochemical index, *MUFA* Mono unsaturated fatty acids, *SFA* Saturated fatty acids

^*^Adjusted for energy intake except energy variable. Low and High are presented as lower and higher adherence of median

### The association between DN and PI score

Odds ratios (OR) and 95% confidence intervals (CI) of DN, from crude and adjusted models, across dietary PI groups are shown in Table 4. There was an inverse relationship between dietary PI and risk of DN (OR = 0.44; 95% CI: 0.25–0.77; *P* = 0.04). After adjusting for potential confounders, the association remained significant, albeit with lower odds of having DN (OR = 0.15; 95% CI: 0.06–0.36; *P* < 0.001).

**Table 4:** Odds ratios and 95% confidence intervals of diabetic nephropathy based on lower and higher adherence of median of dietary phytochemical index among cases and controls

Variables	PI score		β	SE	*P* value
	**Low**	**High**			
**N case/control**	32/69	36/73			
**Crude**	1.00 (ref)	0.44(0.25–0.77)	-0.81	0.28	**0.04**
**Model 1**	1.00 (ref)	0.17(0.08–0.38)	-1.73	0.39	** < 0.001**
**Model 2**	1.00 (ref)	0.15(0.06–0.36)	-1.85	0.42	** < 0.001**

Logistic regression was used. Low and High are presented as lower and higher adherence of median

Odds ratio (95% confidence interval) are shown across lower and higher adherence of median. *SE* Standard error

Model 1: Adjusted for age, body mass index, energy intake, and physical activity

Model 2: Adjusted for model 1 + diabetes duration, cardiovascular diseases history, and drug usage (angiotensin receptor blockers; angiotensin converting enzyme inhibitors, beta-blockers, metformin, sulphonyl urea, and insulin)

## Discussion

To our knowledge, this is the first case–control study designed to assess the relationship between a dietary phytochemical index and the risk of diabetic nephropathy in Iranian women. In the current study, lower levels of FBS were observed in higher adherence of median of PI in the case group. Similar to our study, Aghdam et al., in a cross-sectional study, observed that participants with a higher PI had lower FBG [9]. Also, a longitudinal study reported a significant negative association between FBS level and PI at baseline, but not after 3-years of follow-up [19]. Numerous studies have reported a significant relationship between intake of various phytochemicals and better FBS levels in healthy participants [20] and patients with type 2 diabetes mellitus (T2DM) [21, 22]. However, some studies have suggested no significant association between FBS and PI in healthy subjects was evident [23].

Our results showed that, compared to the control group, case group participants had a lower daily intake of vegetables, fruits, grains, legumes, and olives. In addition, higher intakes of mentioned food groups in the higher adherence of median of dietary PI in controls may have an effective influence in the decreasing risk of DN in control group. No significant relationship was found between PI and lipid profile; however, in contrast to our study, Aghdam et al. [9] reported that a higher intake of phytochemical-rich food may be related to lower LDL and higher HDL levels. Golzarand et al. [24] found that the levels of TC, TG, and HDL were, in the highest quartile of PI, significantly reduced in healthy men, but not in women, after 3 years of follow-up [24]. The difference in characteristics of participants, study design, study sample size, food patterns, and eating habits of people in different countries are likely contributors to the inconsistent results in the literature. Also, the findings of our study suggest that phytochemicals could have a protective effect on DN, as we observed a negative relationship between dietary PI and the risk of DN. In line with our results, in 2020, a systematic review and meta-analysis comparing different dietary patterns, which had several common components including olive oil, whole grains, fruits, nuts, vegetables, and legumes, reported that these components might reduce diabetes and its complications [25]. The relationship and potential effects of dietary phytochemicals on the prohibition of T2DM and hyperinsulinemia have been corroborated in recent studies [26]; however, prior to our study, the relationship between PI and diabetic nephropathy has not previously been determined.

In general, several mechanisms might be involved in the relationship between PI and DN. Phytochemicals have been shown to confer ameliorative effects on diabetes and its complications [27]. It has also been reported that the properties of phytochemicals can ameliorate renal injury and pathologic metabolic alteration via the control of numerous signaling pathways. Additionally, plants are a major source of antioxidants and facilitate nephron conservation through a decrease in oxidative pressure, which consequently helps to control diabetes and its complications [28]. Mono and polyunsaturated fatty acids, and other bioactive compounds containing fiber, tocopherols, phenolic compounds, and phytosterols, have been reported to be beneficial in alleviating inflammation and oxidative stress and in reducing insulin resistance and secretion, which are pathogenic factors in diabetes [29] and diabetic microvascular complications [30]. Phytochemicals can impact carbohydrate metabolism and improve FBS [31] through inhibition of carbohydrate digestion and intestinal glucose absorption, stimulation of insulin secretion from pancreatic β-cells, stimulation of hepatic glycolysis and glycogenesis, antioxidant properties, effect on intracellular signaling pathway, and gene expression [32]. The use of phytochemicals has also been related to reduced mortality and chronic disease risk [33, 34]. However, reduction in dietary fiber (as a rich source of phytochemicals) may influence glycemic control, insulin sensitivity, and augment inflammation [35]. Thus, foods rich in phytochemicals may provide advantages in the inhibition of chronic disease.

Numerous strengths of the current study are worthy of consideration. To our knowledge, this is the first study to have assessed the association between PI and the risk of diabetic nephropathy in a case–control design. In addition, we considered non-calorie phytochemical-rich foods such as tea and spices. However, our study also has some limitations. The case–control nature of the study precludes cause and effect conclusions. Moreover, small errors in the dietary assessment may be present, mostly due to mis-recalling the data and misclassification errors by using FFQ. Another limitation is the lack of control for education in the analysis that might affect our findings. Moreover, our study only included women, thus, results are not generalizable to men.

## Conclusion

In conclusion, we found evidence indicating an inverse relationship between consumption of foods rich in phytochemicals and risk of diabetic nephropathy in a sample of Iranian women. However, to confirm the veracity of these findings, further studies with larger sample sizes are needed.

## Supplementary Information

**Additional file 1. **

## Data Availability

The datasets generated and/or analyzed during the current study are not publicly available as per the rules and regulations of the Community Nutrition Department of Tehran University of Medical Science, but are available from the corresponding author upon request.

## Abbreviations

BMI: Body mass index
PI: Phytochemical index
DN: Diabetic nephropathy
LED: Low energy density
HED: High energy density
PA: Physical activity
WC: Waist circumference
ACR: Albumin per gram of creatinine
T2DM: Type two diabetes mellitus
FBS: Fasting blood sugar
FBG: Blood level of fasting glucose
2HBG: 2 Hour blood glucose
TG: Triglycerides
TC: Total cholesterol
Cr: Serum creatinine
BUN: Blood urea nitrogen
FFQ: Food frequency questionnaire
ARBs: Angiotensin receptor blockers
ACEIs: Angiotensin converting enzyme inhibitors
