# Supplementary figures and images for: High intake of dietary phytochemical index may be related to reducing risk of diabetic nephropathy: a case–control study
Source: BMC Nutr. 2023 Jan 16;9:14. doi: 10.1186/s40795-023-00676-2 (PMC9841724; doi:10.1186/s40795-023-00676-2)

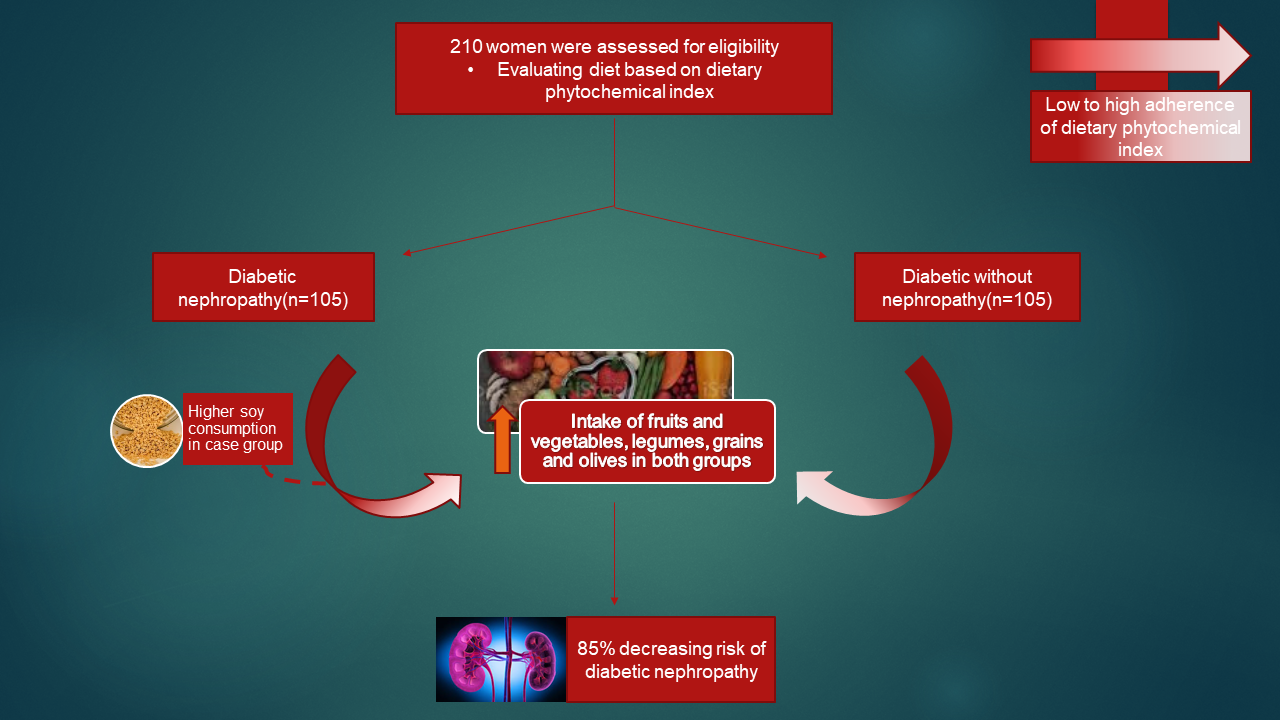

Supplement: Supplementary file 1 — Additional file 1. [file 40795_2023_676_MOESM1_ESM.tif]
